# Supplementary material for: Heat adaptation of phage T7 under an extended genetic code
Source: Virus Evol. 2021 Dec 1;7(2):veab100. doi: 10.1093/ve/veab100 (PMC8923235; doi:10.1093/ve/veab100)
Supplement: veab100_Supp [file veab100_supp.zip › Sequencing_Analysis.zip!/Sequencing_Analysis/A1/evidence/RA_35.html]

BRESEQ :: Evidence


| Read alignment evidence... | | | | | | | | | | | |
| --- | --- | --- | --- | --- | --- | --- | --- | --- | --- | --- | --- |
|  | seq id | position | | ref | new | freq | score (cons/poly) | reads | annotation | genes | product |
| \* | NC\_001604.1 | 28,943 | 0 | C | A | 21.4% | 5538.9 / inf | 2669 | intergenic (–/–) | *–/–* | –/– |
| Reads supporting (aligned to +/- strand):  **ref** base C (1121/966);  **new** base A (286/283);  **total** (1413/1256) | | | | | | | | | | | |
| Fisher's exact test for biased strand distribution *p*-value = 1.55e-01 | | | | | | | | | | | |
| Kolmogorov-Smirnov test that lower quality scores support variant *p*-value = 9.91e-01 | | | | | | | | | | | |

Only 100 of 2690 total aligned reads displayed.  

`AAGACTGGTCGTAACGCTGCGTATCTTGTGGACGATGACGTTATGCAGAAGATAAAAGAGGGTGTCTTCCGTACTCGCGAAGAGATGGAAGAGTATCGCCATAGTCGCCTTCAAGAGGGCGCTAAGGTATACGCTGAGCAGTTCGGCATCGACCCTGAGGACGTTGATTATCAGCGTGGTTTCAACGGGGACATTACCGAGCGTAACATCTCGCTGTATGGTGCGCATGATAACTTCTTGAGCCAGCAAGCTCAGAAGGGCGCTATCATGAACAGCCGAGTGGAACTCAACGGTGTCCTTCAAGACCCTGATATGCTGCGTCGTCCAGACTCTGCTGACTTCTTTGAGAAGTATATCGACAACGGTCTGGTTACTGGCGCAATCCCATCTGATGCTCAAGCCACACAGCTTATAAGCCAAGCGTTCAGTGACGCTTCTAGCCGTGCTGGTGGTGCTGACTTCCTGATGCGAGTCGGTGACAAGAAGGTAACACTTAACGGAGCCACTACGACTTACCGAGAGTTGATTGGTGAGGAACAGTGGAACGCTCTCATGGTCACAGCACAACGTTCTCAGTTTGAGACTGACGCGAAGCTGAACG  >  NC_001604.1/28643‑29243`  

                                                                                                                                                                                                                                                                                                            `|`                                                                                                                                                                                                                                                                                                              
`aaGACTGTGCGTAACGCTGCCGTTCTTGTGGACGATGACGTTATGCAGCAAGTAAAAGAGGGTGTCTTCCGTACTCACGAAGAGATGGAATAGTATCGCCATAGTCGCCTTCAAGAGGGCGCTAAGGTATACGCTGAGCATTTCTGCATCGACCCTGAGTACGGTGATTATCAGAGTGGTTGCAACGGGGACATTACCGAGCGTAACATCTCGCTGTATGGTGCTCATGATAACTTCTTGAGCCAGCAAGCTCAGAAGGGCGCTATCATGAACAGCCGAGTGGAACTCAACGGTGTACTTc                                                                                                                                                                                                                                                                                                              <  1:252626/301‑1 (MQ=255)`  
  `gACTGGTCGTAACGCTGCGTATCTTGTGGACGATGACGTTATGCAGCAGATAAAAAAGGGTGTCTTCCGTACTCGCGAAGAGATGGAAGAGTATCGCCATAGTCGCCTTCAAGAGGGCGCTAAGGTATACGCTGAGCAGTTCGGCATCGACCCTGAGGACGTTGATTATCAGCGTGGTTTCAACGGGGACATTACCGAGCGTAACATCTCGCTGTATGGTGCGCATGATAACTTCTTGAGCCAGCAAGCTCAGAAGGGCGCTATAATGAACAGCCGAGTGGAACTCAACGGTGTCCTTaaa                                                                                                                                                                                                                                                                                                            <  1:113776/301‑1 (MQ=255)`  
   `aCTGGTCGTAACGCTGCGTATCTTGTGGACGATGACGTGATGCAGCAGATAAAAGATGGTGTCTTCCGTACTCGCGAAGAGATGGAAGAGTATCGCCATAGTCGCCTTCAAGAGTGCGCTAAGGTATACGCTGAGCAGTTCGGCATCGACCCTGAGGACGTTGATTATCAGCGTGGTTTCAACGGGGACATTACCGAGCGTAACATCGCGCTGTATGGTGCGCATGATAACTTCTTGAGCCAGCAAGCTCAGAAGGGCGCTATCATGAACAGCCGAGTGGAACTCAACGGTGTCCTTAAAg                                                                                                                                                                                                                                                                                                           <  1:229830/301‑1 (MQ=255)`  
    `cTGGTCTTAACGCTGCGTATATTTTGGACGATTACGTTATGCAGCAGATAAAAGAGGGTGTCTTCCGTACTCGCGAAGAGATGGAAGAGTATCGCCATAGTCGCCTTCAAGAGGGCGCTAAGGTATACGCTGAGCAGTTCTGCATCGACCCTTAGGGCGTTGATTATCAGCGTGGTTTCAACGGGGACATTACCGAGCGTAACATCTCGCTGTATGGTGCGCATGATAACTTCTTGAGCCAGCAAGCTCAGAAGGGCGCTATCATGAACAGCCGAGTGGAACTCAACGGTGTCCTTAAAGa                                                                                                                                                                                                                                                                                                          <  1:176821/301‑1 (MQ=255)`  
     `gggTCGTAACGCTGCGTATCTTGTGGACGAGGAGGGTATGCAGCAGATAAAAGAGGGTGTCTTCCGTACTGGGGAAGAGATGGAAGAGGATCGCCATAGTCGCCTTCAAGAGGGCGCTAAGGTATACGCTGAGCAGTTCGGCATCGACCTTGAGGACGTTGATTATCAGCGTGGTTTCAACGGGGACATTACCGAGCGTAACATCTCGCTGTATGGTGCGAATGATAACTTCTTGAGCCAGCAAGCTCAGAAGGGCGCTATCATGAACAGCCGAGTGGAACTCAACGGTGTCCTTCAAGAc                                                                                                                                                                                                                                                                                                         <  1:40057/300‑1 (MQ=255)`  
      `tgTCGTAACGCTTCGTATCTTGTGGACGATGACGTTATTCAGCAGATAAAAGAGGGTGTCTTCCGTACTCGCTAAGAGCTGCACGAGTATCGCCATAGTCGCCTTCAAGGTGGCGCTCCGGAATACGCTTCTCCGTTCTGCCTCTGCCCTGAGGACGTTTCTTATCAACGTGCATTCAACGGTCACATTACCGAACGTAACATCTCGCTGTATGGTGCGCATGATACCTTCTTGAGCCAGCAAACTCAGAATGCCGCTCTCATAACCATCCGAGTGGAACTCACCAGTGTCCTGCAAGAcc                                                                                                                                                                                                                                                                                                        >  1:419425/2‑301 (MQ=255)`  
       `gTCGTAACGCTGCGTATCTTGTGGACGATGACGTTATGCAGCAGATAAAAGAGGGTGTCTTCCGTACTCGCGAAGAGATGGAAGAGTATCGCCATAGTCGCCTTCAAGAGGGCGCTAAGGTATACGCTGAGCAGTTCGGCATCGACCCTGAGGACGTTGATTATCAGCGTGGTTTCAACGGGGACATTACCGAGCGTAACATCTCGCTGTATGGTGCGCATGATAACTTCTTGAGCCAGCCAGCTCAGAAGGGCGCTATCATGACCAGCCGAGTGGAACTCAACGTTGTCCTTACAGAccc                                                                                                                                                                                                                                                                                                       >  1:127128/1‑301 (MQ=255)`  
           `tAACGCTGCGTATCTTGTGGACGATGACGTTATGCAGCAGATAAAAGAGGGTGTCTTCCGTACTCGCGAAGAGATGGAAGAGTATCGCCATAGTCGCCTTCAAGAGGGCGCTAAGGTATACGCTGAGCAGTTCGGCATCGACCCTGAGGAAGTAGATTATCAGCGTGGTTTCAACGGGGACATTACCGAGCGTAACATCTCGCTGTATGGTGCGCATGATAACTTCTTGAGCCAGCAAGCTCAGAAGGGCGCTATCATGAACAGACGAGTGGAACTAAACGGGGGCCTTTCAGCCCATGat                                                                                                                                                                                                                                                                                                   >  1:80804/1‑301 (MQ=255)`  
             `aCGTTGCTTATCTGGTGTACCATGACGTTATCCATCAGCTAAAAGATGGTGTCTTGCGTCATCGCGAAGAGATGGAAGAGTAGCGCCTTAGTCGCCTTCAAGAGGGCGCTAAGGTATACTCTTAGCAGTTCTGCATCAACCCTGATGACGTTGATTATCAGCGTGGTTTCAACGGGGACATTACCGAGCGTAACATCTCGCTTTATGGTGCGCATGATACCTTCTTGAGCCAGCAAGTTCAGAAGGGCGCTATCATGAACAGCCGAGTGGAACTCAACGGTGTCCTTAAAGACCCTGatat                                                                                                                                                                                                                                                                                                 <  1:258538/301‑1 (MQ=255)`  
               `aagagacaacTTGTGGACGGTGACATTCTGCAGCAGATAAAAGAGGGTGTCTTCCGTACTCGCGAAGGGATGTAAGGGTATCGCCATAGTCGCCTTCAAGAGGGCTCTAAGGTATACTGTGAGCAGTTCGGCATCGACCCTGAGGACGTTGATTATCAGCGTGGTTTCGACGGGGACATTACCGAGCGTAACATCTCGCTGTATGGTGCGCATGATAACTTCTTGAGCCAGCAAGCTCAGAATGGCGCTATCATGAACAGCCGAGTGGACCTCAACGGTGTCCTTCAAGACCCTGATAtgc                                                                                                                                                                                                                                                                                               <  1:220698/292‑1 (MQ=255)`  
                       `tCTTGTGGACGATGACGTTATGCAGCAGATAAAAGAGGGTGTCTTCCGTACTCGCGAAGAGATGGAAGAGTATCGCCATAGTCGCCTTCAAGAGGGCGCTAAGGTATACGCTGAGCAGTTCGGCATCTACCCTGAGGACGTTTATTATCAGCGTGGTTTCAACGGGGACATTACCGAGCGTAACATCTCGCTGTATGGTGCGCATGATAACTTCTTGAGCCAGCAAGCTCAGAAGGGCGCTATCATGAACAGCCGAGTGGAACTCAACGGTGTCCTTCAAGACCCTGATATGCTGcgtcgt                                                                                                                                                                                                                                                                                       <  1:194707/301‑1 (MQ=255)`  
                       `tCTTGTGGACGATGACGTTATGCAGCAGATAAAAGAGGGTGTCTTCCGTACTCGCGAAGAGATGGAAGAGTATCGCCATAGTCGCCTTCAAGAGGGCGCTAAGGTATACGCTGAGCAGTTCGGCATCGACCCTGAGGACGTTGATTATCAGCGTGGTTTCAACGGGGACATTACCGAGCGTAACATCTCGCTGTATGGTGCGCATGATCACTTCTTGAGCCAGCAAGCTCAGAAGGGCGCTATCATGAACAGCCTAGTGGCACTCAACGGTGGCCTTCCAgctgtctcttatacccaactc                                                                                                                                                                                                                                                                                       >  1:488448/1‑281 (MQ=255)`  
                               `aCGATGACGTTATGCAGCAGATAAAAGAGGGTGTCTTCCGTACTCGCGAAGAGATGGAAGAGTATCGCCATAGTCGCCTTCAAGAGGGCGCTAAGGTATACGCTGAGCAGTTCGGCATCGACCCTGAGGACGTTGATTATCAGCGTGGTTTCAACGGGGACATTACCGAGCGTAACATCTCGCTGTATGGTGCGCATGATAACTTCTTGAGCCAGCAAGCTCAGAAGGGCGCTATCATGAACAGCCGAGTGGAACTCAACGGTGTCCTTCAAGACCCTGATATGCTGCGTCGTCCAGActc                                                                                                                                                                                                                                                                               <  1:395709/301‑1 (MQ=255)`  
                                  `aTGACGTTATTCAGCAGATAAAAGAGGGTGTCTTCCGTGCTCGCGAAGAGATGGAAGAGTATCGCCATAGTCGCCTTCAAGAGGGCGCTAAGGTATACGCTGAGCAGTTAGGCATCGACCCTGAGGACGTTGATTATCAGCGTGGTTTCAACGGGGACATTACCGAGCGTAACATCTCGCTGTATGGTGCGCATGAAAACTTCTTGAGCAAGCAAGCTCAGAAGGGCGCTATCATGAACAGCCGAGTGGAACTCAACGGTGTCCTTCACGACCCCGATCTGCTGCGTCGTCCCGACTctgt                                                                                                                                                                                                                                                                            >  1:350337/1‑300 (MQ=255)`  
                                               `gcagaTAAAAGAGGGTGTCTTCCGTACTCGCGAAGAGATGGAAGAGTATCGCCATAGTCGCCTTCAAGAGGGCGCTAAGGTATACGCTGAGCAGTTCGGCATCGACCCTGAGGACGTTGATTATCAGCGTGGTTTCACCGGGGACATTACCGAGCGTAACATCTCGCTGTATGGTGCGCATGATAACTTCTTGAGCCAGCAAGCTCAGAAGGGCGCTATCATTAACAGCCGAGTGGAACTCAACGGTGTCCTTAAAGACCCTGATATGCTGCGTCGTCCAGACTCTGCTGACTTCTTTgag                                                                                                                                                                                                                                                               >  1:233344/3‑301 (MQ=255)`  
                                                `aaaataaaagaggtggatatacGTACGTGCGAAGAGATGTAAGAGTATCGACATAGTCGACGTAAAGAGGGAGCTAAGGTATACGCTGAGCAGTTCGGCATGGACCTTGAGGACGATGATTATCCGAGTGGTTCCAACGGAGACATTACCGAGCGTAACATATCGCTGGATGGTGTGGATGATAACTTCTTGAGCCAGCAAGCTCAGAAGGGCGCTATCATGAACAGCCGAGTGGAACTCAACGGTGTCCTTCAAGACCCTGATATGCTGCGTCATCCAGACTCTGCTGACTTCTTTgaga                                                                                                                                                                                                                                                              <  1:62023/280‑1 (MQ=255)`  
                                                  `gaTAAAAGAGGGTGTCTTCCGTACTCGCGAAGAGATGGAAGAGTATCGCCATAGTCGCCTTCAAGAGGGCGCTAAGGTATACGCTGAGCAGTTCGGCATCTACCCTGAGGACGTTGATTATCAGCGTGGTTTCAACGGGGACATTACCGAGCGTAACATCTCGCTGTATGGTGCGCATGATAACTTCTTGAGCCCGCAAGCTCAGAAGGGCGCTATCATGAACAGCCGAGTGGAACTCAACGGTGTCCTTAAAGACCCTGATATGCTGCGTCGTCCAGACTCTGCTGACTTCTTTGAGAAg                                                                                                                                                                                                                                                            >  1:157733/1‑301 (MQ=255)`  
                                                  `gaTAAAAGAGGGTGTCTTCCGTACTCGCGAAGAGATGGAAGAGTATCGCCATAGTCGCCTTCAAGAGGGCGCTAAGGTATACGCTGAGCAGTTCGGCATCGACCCTGAGGACGTTGATTATCAGCGTGGTTTCAACGGGGACATTACCGAGCGTAACATCTCGCTGTATGGTGCGCATGATAACTTCTTGAGCCAGCAAGCTCAGAAGGGCGCTATCATTAACAGCCGAGTGGAACTCAACGGTGTCCTTCAAGACCCTGATATGCTGCGTCGTccctgtcccttatacacatctccgagc                                                                                                                                                                                                                                                            >  1:276859/1‑276 (MQ=255)`  
                                                       `aaGAGGGGTACTGCCGTACTCGCGAAGAGATGGAAGAGTATCTCCATAGTCGTCTTCAAGAGGGCGCTAAGGTATACGCTGAGTATTTCGGCATCGACCCTGAGGACGTTGATTATCAGCGTGGTTTCAACTGGGACATTACCGAGCGTAACATCTCGCTGTATGGTGCGCATGATAACTTCTTTAGCCAGCAAGCTCAGAAGGGCGCTATCATGAACAGCCGAGTGGAACTCAACGGTGTCCTTCAAGACCCTGATATGCTGCGTCGTCCAGACTCTGCTGACTTCTTTGAGAAGTatat                                                                                                                                                                                                                                                       <  1:67639/301‑1 (MQ=255)`  
                                                         `gagGGTGTCTTCCGGACTCGCGAAGAGATGGAAGAGTATCGCCATAGTCGCCTTCAAGAGGGCGCTAAGGTATACGCTGATCAGTTCGGCATCGACCCTGAGGACGTTGATTATCAGCGTGGTTTCCACGGGGACATTACCGAGCGTAACATCTCGCTGTATGGTGCGCATGATAACTTCTTGAGCCAGCAAGCTCAGAAGGGCGCTATCATGAACAGCCGAGTGGAACTCAACGGTGTCCTTCAAGACCCTGATATGCTGCGTCGTCCAGACTCTGCTGACTTCTTTGAGAAGTATATCg                                                                                                                                                                                                                                                     <  1:109031/301‑1 (MQ=255)`  
                                                           `gggggacTTGCGTACTCGCGAAGAGATGGAAGAGTATCGCCATAGTCGCCTTAAAGAGGGCGCTAAGGTATACGCTGAGCAGGTCGGCAGCGACCCTGAGGACGTTGATTATCAGCGTGGTTTCAACGGGGACATTACCGAGCGTAACATCTCGCTGTAGGGTGCGCATGATAACTTCTTGAGCCAGCAAGCTCAGGAGGGCGCTATCATTAACAGCCGAGTGGAACTCAACGGTGTCCTTCAAGACCCTGATATGCTGCGTCGTCCAGACTCTGCTGACTTCTTTGAGAAGTATATCGAc                                                                                                                                                                                                                                                   <  1:145835/295‑1 (MQ=255)`  
                                                            `ggTGTCTTCCGTACTCTCGAAGAGATGGAAGAGTATCGCCATAGTCGCCTTCAAGAGGGCGCTAAATTATACGCTGAGCAGTTCGGCATCGACCCTGAGGACGTTGATTATCATCGTGGTTTCAACGGGGACATTACCGAGCGTAACATCTCGCTGTATGGTGCGCATGATAACTTCTTGAGCCAGCAAGCTCAGAAGGGAGCTATCATGAACAGCCGAGTGGCACTCAACGGTGTCCTTAAAGACCCTGATATTCTTCGTCGTCCAGACTCTGCTGACTTCTTTtagctgtctcttatac                                                                                                                                                                                                                                                  >  1:44116/1‑288 (MQ=255)`  
                                                             `gtgtCTTCCGTACTCGCGAAGAGATGGAAGAGTATCGCCATAGTCGCCTTCAAGAGGGCGCTAAGGTATACGCTGAGCAGTTCGGCATCGACCCTGAGGACGTTGATTATCAGCGTGGTTTCAACTGGGACATTACCGAGCGTAACATCTCGCTGTATGGTGCGCATGATAACTTCTTGAGCCAGCAAGCTCAGAAGGGCGCTATCATGAACAGCCGAGTGGAACTCAACGGTGTCCTTAAAGACCCTGATATGCTGCGTCGTCCAGACTCTGCTGACTTCTTTGAGAAGTATATCGACaa                                                                                                                                                                                                                                                 >  1:136327/1‑301 (MQ=255)`  
                                                                 `cTTCCGTACTCGCGAAGAGATGGAAGAGTATCGCCATAGTCGCCTTCACGAGGGCGCTAAGGTATACGCTGAGCAGTTCCCCATCGACCCTGAGGACGTTGATTATCAGCGTGGTTTCAACGGGGACATTACCGAGCGTAACATCTCTATGTATGGTGCGCTTGATAACTTCTTGAGCCCGCACGCTCACAAGGGCGCTATCATGACCAGCCGAGTGTAACTCAACGGTGTCCTTCAAGACCCTGATATGCTGCGTCGTCCAGACTCTGCTGACTTCTTTGAGACGTATATTTACAACGGt                                                                                                                                                                                                                                             >  1:18484/1‑301 (MQ=255)`  
                                                                     `gagacaggcgAAGAGATGGAAGAGTATCGCCATAGTCGCCTTCAAGAGGGCGCTAAGGTATACGCTGAGCAGTTCGGCATCGACCCTGAGGACGTTGATTATAAGCGTGGTTTCAACGGGGACATTACCGAGCGTAACATCTCGCTGTATGGTGCGAATGATAACTTCTTGAGCCAGCAAGCTCAGAAGGGCGCTATCATGAACAGCCGAGTGGAACTCAACGGTGTCCTTCAAGACCCTGATATGCTGCGTCGTCCAGACTCTGCTGACTTCTTTGAGAAGTATATCGACAACGGTCTgg                                                                                                                                                                                                                                         <  1:181409/294‑1 (MQ=255)`  
                                                                       `gagtCGCGAAAGGATGTAATCTTATCTCCATAGTCGCCTTCAAGAGGGCGCTAAGTTATTCGCTGAGCAGTTCGGCATCGACCCTGAGGACGTTGATTATCAGCGTGGTTTCAACGGGGACATTACCGAGCGTAAAATCTCGCTGTATTGTGCGCATGATAACTTCTTGAGCCAGCAAGCTCAGAAGGGCGCTATCATGAACAGCCGAGTGGAACTCAACGGTGTCCTTCAAGACCCTGATATGCTGCGTCGTCCAGACTCTGCTGACTTCTTTGAGAAGTATATCGACAACGGTCTGGtt                                                                                                                                                                                                                                       <  1:295633/298‑1 (MQ=255)`  
                                                                        `aCTCGCGAAGAGATGGAAGAGTATCGCCATAGTCGCCTTCAAGAGGGCGCTAAGGTATACGCTGAGCAGTTCGGCATCGACCCTGAGGACGTTGATTATCAGCGTGGTTTCAACGGGGACATTACCGAGCGTAACATCTCGCTGTATGGTGCGCATGATAACTTCTTGAGCCAGCAAGCTCAGAAGGGCGCTATCATGAACAGCCGAGTGGAACCCAACGGTGTCCTTCAAGACCCTGATATGCTGCGTCGTCCAGACTCTGCTGACTTCTTTGAGAAGTATATCGACAACGGTCTGGTTa                                                                                                                                                                                                                                      <  1:224626/301‑1 (MQ=255)`  
                                                                           `cgcgAAGAGATGGAAGAGTATCGCCATAGTCGCCTTCAAGAGGGCGCTAAGGTATACGCTGAGCAGTTCGGCATCGACCCTGAGGACGTTGATTATCAGCGTGGTTTCAACGGGGACATTACCGAGCGGAACATCTAGCTGTATGGTGCGCATGATAACTTCTTGAGCCAGAAAGCTCAGAAGGGCGCTATCCTGAACAGCCGAGTGGAACTCAACGGTGTACTTAAAGACCCTGATATGCTGCGTCGTCAAGACTCTGCTGACTACTTTGAGAAGTATATCGACAACGTGCGGGTTAcgg                                                                                                                                                                                                                                   >  1:95901/1‑299 (MQ=255)`  
                                                                            `gcgAAGAGCTGGAAGAGTATCTCCATAGTCGCCTGCAAGAGGGCGCTAATGTATACGCTGAGCAGTTCGGCATCGACCATGAGGACTTTGATTATCAGCTTGGTTTCAACGGGGACATTACCGAGCGTAACCTCTCGCTGTATGTGGCGCATGATAACTTCTTGAGCCAGCAAGCTCAGAAGGGCGCTATCATGAACAGCCGAGTGGAACTCAACGGTGTCCTTCAAGACCCTGATATGCTGCGTCGTCCAGACTCTGCTGACTTCTTTGAGAAGTATATCGACAACGGTCTGGTTACTgg                                                                                                                                                                                                                                  <  1:344802/301‑1 (MQ=255)`  
                                                                                         `gacagaTCGCCATAGTCGCCTTCAAGAGGGCGCTAAGGTAGACGCTGAGCAGTTCGGCATCGACCCTGAGTACGTTGATTATCAGCGTGGTTTCAACGGGGACATTACCGAGCGTAACATCTCGCTGTATGGTGCGCATGATAACTTCTTGAGCCAGCAAGCTCAGAAGGGCGCTATCATGAACAGCCGAGTGGAACTCAACGGTGTCCTTCAAGACCCTGATATGCTGCGTCGTCCAGACTCTGCTGACTTCTTTGAGAAGTATATCGACAACGGTCTGGTTACTGGCGCAATCCCATCt                                                                                                                                                                                                                     <  1:340677/296‑1 (MQ=255)`  
                                                                                         `agagTATCGCCCTAGTCGCCTCTAAGAGCTCTCTAATCTAACTGCTAACGAGTTCTGCATCGACCCTGAGCACGTTAATTATCATTGTCGTTTGAACGGTGTCTTTAACAGGGGTAACATCTAGATGTTTGGTCGGTAGTTTAACTGCTAGAACCGTCAAGCTTAGAAGGGCGTTAGCATGAACAGCCTCGTGGAACTCAACAGTGCTTTTCAAGAACCTGATATGCTGCGTCGTCCAGACTTTGCTGACTTCTTGTAGAAGTATATCTACAACGGTCTGGTTACTGGCGCAATCCCATCt                                                                                                                                                                                                                     <  1:336336/301‑1 (MQ=255)`  
                                                                                              `agcGCCATAGGCGGCTGCAAGAGGGAGCTAAGGTATACGCTGAGCAGTGCGGCATCGACCCTGAGGACGTTGAGTATCAGCGTGGGTTTAACGGGGACAGTACCGAGCGTAACATCTCGCTGTATGGTGCTCATGATAACTGCTTGAGCCAGCAAGCTCAGAAGGGCGCTATCATGAACAGCCGAGTGGAACTCAACGGTGTCCTTCAAGACCCTGATATGCTGCGTCGTCCAGACTCTGCTGACTTCTTTGAGAAGTATATCGACAACGGTCTGGTTACTGGCGCAATCCCATCTGATGc                                                                                                                                                                                                                <  1:53106/299‑1 (MQ=255)`  
                                                                                               `tCGCCTTAGTCGCCTTCAAGAGGGCGCTAAGGTATACGCTGAGCAGTTCGGCATCGACCCTGAGGACGTTGATTATCAGCGTGGTTTCAACGGGGACATTACCGAGCGTAACATCTCGCTGTATGGTGCGCATGATAACTTCTTGAGCCAGCAAGCTCAGAAGGGCGCTATCATGAACAGCCGAGTGGAACTCAACGGTGTCCTTCAAGACCCTGATATGCTGCGTCGTCCAGAATCTGCTGACTTCTTTGAGAAGTCTCTCGACAACGGTCCGGTTACTGGCGCAATCCCATCCgctgct                                                                                                                                                                                                               >  1:363971/1‑301 (MQ=255)`  
                                                                                                `cGCCAGAGTCGCCGTCAAGGGGTCGCTAAGTTATACGTGGGGCTGTTCGGCCTCGACCCTGAGGTCGTTGATTATCTACGTGGTTTCAACGTGGACATTACCGGGCGTAACATCTCGCTGTATAGTTCGCATGCTAACTTCTTGAGCCAGCAAGCTCAGAAGGGCGCTATCATGAACGGCCGAGTGTAACTCAACGGTGTCCTTCAAGACCCTGATATGCTGCGTCGTCCAGACTCTGCTGACTTCTTTGAGAAGTATATCTACAACGGTCTGGTTACTGGAGCAATCCCATCTGATGCTc                                                                                                                                                                                                              <  1:217542/301‑1 (MQ=255)`  
                                                                                                     `tAGTCGCCTTCAAGAGGGCGCTAAGGTATACGCTGAGCAGTTCGGCATCGACCCTGAGGACGTTGATTATCAGCGTGGTTTCAACGGGGACATTACCGAGCGTAACATCTCGCTGTATGGTGCGCATGATAACTTCTTGAGCCAGCAAGCTCAGAAGGGCGCTATCATGAACAGCCGAGTGGAACTCAACGGTGTCCTTCAAGACCCTGATATGCTGCGTCGTCCAGACTCTGCTGACTTCTTTGAGAAGTATATCGACAACGATCTGGTTACTGGCGCAATCCCATCTGATGCTCAAGcc                                                                                                                                                                                                         <  1:273166/301‑1 (MQ=255)`  
                                                                                                       `gTCGACTTAAAGAGGTCGCTAAGGTATACGCTGAGCAGTTCGGAAGCGACCCTGAGGACGTTGATTATCAGCGTGGTTTCAACGGGGACAATACCAAGCGTAACATCTCGCTGTATGGTTCGCATGATAACTTCTTGAGCCAGCAAGCTCAGAAGGGCGCTATCATGAACAGCCGAGTGGAACTCAACGGTGTCCTTCAAGACCCTGATATGCTGCGTCGTCCAGACTCTGCTGACTTCTTTGAGAAGTATATCGACAACGGTCTGGTTACTGGCGCAATCCCATCTGATGCTCAAGCcac                                                                                                                                                                                                       <  1:306420/301‑1 (MQ=255)`  
                                                                                                         `ggagatgtgtataagagtcaGGTATACGCTGAGCAGTTCGGGATCGACCCTGAGGACGGTGATTATCAGCGTGGTTTCAACGGGGACATTACCGAGCGTAACATCTCGCTGTATGGTGCGTATGATAACTTCTTGAGCCAGCAAGCTCAGAAGGGCGCTATCATGAACAGCATAGTGGAACTCAACGGTGTCCTTCAAGACCCTGATATGCTGCGTCGTCCAGACTCTGCTGACTTCTTTGAGAAGTATATCGACAACGGTCTGGTTACTGGCGCAATCCCATCTGATGCTCAAGCcacac                                                                                                                                                                                                     <  1:451284/282‑1 (MQ=255)`  
                                                                                                         `ggCCTTCAAAGAGGTGATAAGATATACGCTTGGCAGTTCGTCATCGACCCTGAGGACGTTGATTATCAGCGTGGTTTCAACGGGGACATTACCGAGCGTAACATCTCGCTGTATTGTGCGCATGATAACTTCTTGAGCCAGCAAGCTCAGAAGGGAGCTATCATGAACAGCCGAGTGGAACTCAACGGTGACCTTCAAGACCCTGATATGCTGCGTCGTCCAGCCTCTGCTGACTTCTTTGAGAAGTATATCGACACCGGTCTGGTTACTGGCGCAATCCCATCTGATGCTCAAGCcacac                                                                                                                                                                                                     <  1:508500/300‑1 (MQ=255)`  
                                                                                                           `ggggctcggagatgtggataagagacaggaggagTTCGGCATCGACCCGGAGGACGTTGATTATCAGCGTGGTTTCAACGGGGACATTACCGAGCGTAACATCTCGCTGTATGGTGCGCATGATAACTTCTTGAGCCAGCAAGCTCAGAAGGGCGCTATCATGAACAGCCGAGTGGAACTCAACGGTGTCCTTCAAGACCCTGATATGCTGCGTCGTCCAGACTCTGCTGACTTCTTTGAGAAGTATATCGACAACGGTCTGGTTACTGGCGCAATCCCATCTGATGCTCAAGCCACACAg                                                                                                                                                                                                   <  1:356052/273‑1 (MQ=255)`  
                                                                                                           `ccTTCAAGAGGGCGCGAAGTTATCCGCTGAGCAGTTCGGCATCGACCCTGAGGACGTTGATTATCAGCGTGGTTTCAACGGGGACATTACCGAGCGTAACATCTCGCTGTATGGTGCGCATGATAACTTCTTGAGCCAGCAAGCTCAGAAGGGCGCTATCATGAACAGCCGAGTGGAACTCAACGGTGTCCTTCAAGACCCTGATATGCTGCGTCGTCCAGACTCTGCTGACTTCTTTGAGAAGTATATCGACAACGGTCTGGTTACTGGCGCAATCCCATCTGATGCTCAAGCCACACAg                                                                                                                                                                                                   <  1:201706/301‑1 (MQ=255)`  
                                                                                                            `atgtggataagagacagggTATACGCTGAGCAGTTCGGCATCGACCCTGAGGACGTGTATTATCAGCGTTGTTTCAACGGGGACATTACCGAGCGTAACATCTCGCTGTATGGTGCGCATGATAACTTCTTGAGCCAGCAAGCTCAGAAGGGCGCTATCATGAACAGCCGAGTGGAACTCAACGGTGTCCTTAAAGACCCTGATATGCTGCGTCGTCCAGACTCTGCTGACTTCTTTGAGAAGTATATCGACAACGGTCTGGTTACTGGAGCAATCCCATCTGATGCTCAAGCCACACAGc                                                                                                                                                                                                  <  1:100330/284‑1 (MQ=255)`  
                                                                                                                   `ataagagacaGGTATACGCTGAGCAGTTCGGCATCGACCCTGAGGACGTTGATTATCAGCGTGGTTTCAACGGGGACATTACCGAGCGTAACATCTCGCTGTATGGTGCGCATGATAACTTCTTGAGCCAGCAAGCTCAGAAGGGCGCTATCATTAACAGCCGAGTGGAACTCAACGGTGTCCTTCAAGACCCTGATATGCTGCGTCGTCCAGACTCTGCTGACTTCTTTGAGAAGTATATCGACAACGGTCTGGTTACTGGCGCAATCCAATCTGATGCTCAAGCCACACAGCTTATAAg                                                                                                                                                                                           <  1:408203/292‑1 (MQ=255)`  
                                                                                                                    `gggCGCTAAGGTATACGCTGAGCAGTTCGGCATCGACCCTGAGGACGTTGATTATCAGCGTGGTTTCAACGGGGACATTACCGAGCGTAACATCTCGCTGTATGGTGCGCATGATAACTTCTTGAGCCAGCAAGCTCAGAAGGGCGCTATCATGAACAGCCGAGTGGAACTCAACGGTGTCCTTAAAGACCCTGATATGCTGCGTCGTCCAGACTCTGCTGACTTCTTTGAGAAGTATATCGACAACGGTCTGGTTACTGGCGCAATCCCATCTGATGCTCAAGCCACACAGCTTATAAGc                                                                                                                                                                                          <  1:24162/301‑1 (MQ=255)`  
                                                                                                                     `agagacagggTATACGCTGAGCAGTTCGGCATCGACCCTGAGGACGTTGATTATCAGCGTGGTTTCAACGGGGACATTACCGAGCGTAACATCTCGCTGTATGGTGCGCATGATAACTTCTTGAGCCAGCAAGCTCAGAAGGGCGCTATCATGAACAGCCGAGTGGAACTCAACGGTGTCCTTCAAGACCCTGATATGCTGCGTCGTCCAGACTCTGCTGACTTCTTTGAGAAGTATATCGACAACGGTCTGGTTACTGGCGCAATCCCATCTGATGCTCAAGCCACACAGCTTATAAGcc                                                                                                                                                                                         <  1:331486/293‑1 (MQ=255)`  
                                                                                                                      `gcgcTAAGGTATACGCTGAGCAGTTCGGCATCGACCCTGAGGACGTTGATTATCAGCGTGGTTTCAACGGGGACATTACCGAGCGTAACATCTCGCTGTATGGTGCGCATGATAACTTCTTGAGCCAGCAAGCTCAGAAGGGCGCTATCATTAACAGCCGAGTGGAACTCAACGGTGTCCTTCAAGACCCTGATATGCTGCGACGTCCAGGCTATGATGAATTCTTTGAGAAGTATAACGACAACGGGATGGTTACTGGCGCAAGTCCAACTGATGCGCAAGCCACACAGATTATAAGctt                                                                                                                                                                                        >  1:301502/1‑299 (MQ=255)`  
                                                                                                                         `agaaGGTATACGCTGAGCATTTCGGCATCGACCCTGAGGACGTTGATTATCAGCGTGGTTTCAACGGGGACATTACCGAGCGTAACATCTCGCTGTATGGTGCGCATGATAACTTCTTGAGCCAGCAAGCTCAGAAGGGCGCTATCATGAACAGCCGAGTGGAACTCAACTGTGTCCTTAAAGACCCTGATATGCTGCGTCGTCCAGACTCTGCTGACTTCTTTGAGAAGTATATCGACAACGGTCTGGTTACTGGCGCAATCCCATCTGATGCTCAAGCCACACAGCTTATAAGCCAAGc                                                                                                                                                                                     <  1:122094/299‑1 (MQ=255)`  
                                                                                                                           `aaGGTATACGCTGAGCAGTTCGGCATCGACCCTGAGGACGTTGATTATCAGCGTGGTTTCAACGGGGACATTACCGAGCGTAACATCTCGCTGTATGGTGCGTATGATAACTTCTTGAGCCAGCAAGCTCAGAAGGGCGCTATCATGAACAGCCGAGTGGAACTCAACGGTGTCCTTCAAGACCCTGATATGCTGCGTCGTCCAGACTCTGCTGACTTCTTTGAGAAGTATATCGACAACGGTCTGGTTACTGGCGCAATCCCATCTGATGCTCAAGCCACACAGCTtatctgtctcttat                                                                                                                                                                                   >  1:139301/1‑290 (MQ=255)`  
                                                                                                                             `ggTATACGCTGAGCAGTTCGGCATCGACCCTGAGGACGTTGATTATCAGCGTGGTTTCAACGGGGACATTACCGAGCGTAACATCTCGCTGTATGGTGCGCATGATAACTTCTTGAGCCAGCAAGCTCAGAAGGGCGCTATCATTAACAGCCGAGTGGAACTCAACGGTGTCCTTCAAGACCCTGATATGCTGCGTCGTCCAGACTCTGCTGACTTCTTTGAGAAGTATATCGACAACGGTCTGGTTACTGTCGCAATCCCATCTGATGCTCAAGCCACACAGCTTATAAGCCAAGCGTTc                                                                                                                                                                                 >  1:91569/1‑301 (MQ=255)`  
                                                                                                                             `ggTATACGCTGAGCAGTTCGGCATCGACCCTGAGGACGTTGATTATCAGCGTGGTTTCAACGGGGACATTACCGAGCGTAACATCTCGCTGTATGGTGCGCATGATAACTTCTTGAGCCAGCAAGCTCAGAAGGGCGCTATCATGAACAGCCGAGTGGAACTCAACGGTGTCCTTCAAGACCCTGATATGCTGCGTCGTCCAGACTCTGCTGACTTCTTTGAGAAGTATATCGCCAACGGTCTGGTTACTGGCGCAATCCCATCTGATGCTCAAGCCACACAGCTTATAAGCCAAGCGTTc                                                                                                                                                                                 >  1:190035/1‑301 (MQ=255)`  
                                                                                                                             `ggTATACGCTGAGCAGTTCGGCATCGACCCTGAGGACGTTGATTATCAGCGTGGTTTCAACGGGGACATTACCGAGCGTAACATCTCGCTGTATGGCGCGCATGATAACTTCTTGAGCCAGCAAGCTCAGAAGGGCGCTATCATGAACAGCCGAGTGGAACTCAACGGTGTCCTTAAAGACCCTGATATGCTGCGTCGTCCAGACTCTGCTGACTTCTTTGAGAAGTATATCGCCAACGGTCTGGTTACTGGCGCAATCCCATCTGATGCTCAAGCCACACAGCTTATAAGCCAAACGttt                                                                                                                                                                                 >  1:320634/1‑300 (MQ=255)`  
                                                                                                                                  `aCGCTGAGCAGTTCGGCATATACCCTGAGGACGTTGAGTATCAGCGTGGGTTCAACGGGGACATTACCGAGCGTAACATCTCGCTGTATGGTGCGCATGATAACTTCTTGAGCCAGCAAGCTCAGAAGGGCGCTATCATGAACAGCCGAGTGGAACTCAACGGTGTCCTTCAAGACCCTGATATGCTGCGGCGTCCAGACTCTGCTGACTTCTTTGAGAAGTATATCGACAACGGTCTGGTTACTGGCGCAATCCCATCTGATGCTCAAGCCACACAGCTTATAAGCCAAGCGTTCAGTGa                                                                                                                                                                            <  1:327657/301‑1 (MQ=255)`  
                                                                                                                                      `tGAGCAGTTCGGCATCGACCCTGAGTACGTTGATTATCAGCGTGGTTTCAACGGGGACATTACCGAGCGTAACATCTCGCTGTATGGTACGCATGATAACTTCTTGAGCCAGCAAGCTCAGAAGGGCGCTATCATGAACAGCCGAGTGGAAATCAACGGTGTCCTTCAAGACCCTGATATGCTGCGTCGTCCAGACTCTGCTGACTTCTTTGAGAAGTATATCGACAACGGTCTGGTTACTGGCGCAATCCCATCTGATGCTCAAGCCACACAGCTTATAAGCCAAGCGTTCAGTGACGCt                                                                                                                                                                        <  1:378400/301‑1 (MQ=255)`  
                                                                                                                                        `acaggTTTGGCATCGACCCTGAGGACGTTGATTATCAGCGTGGTTTCAACGGGGACATTACCGAGCGTAACATCTCGCTGTATGGTGCACATGATAACTTCTTGAGCCAGCAAGCTCAGAAGGGCGCTATCATGAACAGCCGAGTGGAACTCAACGGTGTCCTTCAAGACCCTGATATGCTGCGTCGTCCAGACTCTGCTGACTTCTTTGAGAAGTATATCGACAACGGTCTGGTTACTGGCGCAATCCCATCTGATGCTCAAGCCACACAGCTTATAAGCCAAGCGTTCAGTGACGCTTc                                                                                                                                                                      <  1:360469/297‑1 (MQ=255)`  
                                                                                                                                           `aGTTCGGCATCGACCCTGAGGACGTTGATTATCAGCGTGGTTTCAACGGGGACATTACCGAGCGTAACATCTCGCTGTATGGTGCGCATGATAACTTCTTGAGCCAGCAAGCTCAGAAGGGCGCTATCATGAACAGCCGAGTGGAACTCAACGGTGTCCTTAAAGACCCTGATATGCTGCGTCGTCCAGACTCTGCTGACTTCTTTGAGAAGTATATCGACAACGGTCTGGTTACTGGCGCAATCCCATCTGATGCTCAAGCCACACAGCTTATAAGCCAAGCGTTCAGTGACGCTTCTAg                                                                                                                                                                   >  1:160904/1‑301 (MQ=255)`  
                                                                                                                                            `gTTCGGCATCGACCCTGAGGACGTAGATTATCATCGTGGTTTCAACGGGGATATTACCGAGCGTAACATCTCGCTGGATGGTGCGCATGATAACGTCTTGAGCCCGCAAGCTCAGAAGGGCGCTATCATTAACAGCCGAGTGGAACTCAACGGTGTCCTTCAAGACCCTGATATGCTGCGTCGTCCAGACTCTGCTGACTTCTTTGAGAAGTATATCGACAACGGTCTGGTTACTGGCGCAATCCCATCTGATGCTCAAGCCACACAGCTTATAAGCCAAGCGTTCAGTGACGCTTCTAGc                                                                                                                                                                  <  1:246353/301‑1 (MQ=255)`  
                                                                                                                                             `ttCGGCATCGACCCTGAGGACGTTGATTATCAGCGTGGTTTCAACGGGGACATTACCGAGCGTAACATCTCGCTGTATGGTGCGCATGATAACTTCTTGAGCCAGCAAGCTCAGAAGGGCGCTATCATGAACAGCCGAGTGGAACTCAACGGTGTCCTTAAAGACCCTGATATGCTGCGTCGTCCAGACTCTGCTGACTTCTTTGAGAAGTATATCGACAACGGTCTGGTTACTGGCGCAATCCCATCTGATGCTCAAGCCACACAGCTTATAAGCCAAGCGTTCAGTGACGCTTCTAGcc                                                                                                                                                                 <  1:478785/301‑1 (MQ=255)`  
                                                                                                                                                      `gACCCTGAGAACGTTGTTTATCAGCGTGGTTTCAACGGGGACATTACCGAGCGTAACATCTCGCTGTATGGTGCGCATGATAACTTCTTGAGCCAGCAAGCTCAGAAGGGCGCTATCATGAACAGCCGAGTGGAACTCAACGGTGTCCTTCAAGACCCTGATATGCTGCGTCGTCCAGACTCTGCTGACTTCTTTGAGAAGTATATCGACAACGGTCTGGTTACTGGCGCAATCCCATCTGATGCTCAAGCCACACAGCTTATAAGCCAAGCGTTCAGTGACGCTTCTAGCCGTGCtggtg                                                                                                                                                        <  1:117660/301‑1 (MQ=255)`  
                                                                                                                                                              `ggACGTTGATTATCAGCGTGGTTTCAACGGGGACATTACCGAGCGTAACATCTCGCTGTATGGTGCGCATGATAACTTCTTGAGCCAGCAAGCTCAGAAGGGCGCTATCATGAACAGCCGAGTGGAACTCAACGGTGTCCTTCAAGACCCTGATATGCTGCGTCGTCCAGACTCTGCTGACTTCTTTGAGAAGTATATCGACAACGGTCTGGTTACTGGCGCAATCCCATCTGATGCTCAAGCCACACAGCTTATAAGCCAACCGTTCAGTCACGCTTCTAGCCGTGCTGGTGGTCCTgcc                                                                                                                                                >  1:197950/1‑299 (MQ=255)`  
                                                                                                                                                                  `gTTGATTATCAGCGTGGTTTCAACGGGGACATTACCGAGCGTAACATTTCGCTGTATGGTGCGCATGATAACTTCTTGAGCCAGCAAGCTCAGAAGGGCGCTATCATGAACAGCCGAGTGGAACTCAACGGTGTCCTTCAAGACCCTGATAGGCTGCGTCGTCCAGACTCCGCTGTCTTCTTTTAGAAGTATATCGACAACGGTCTGGTTACTGGCGCAATCCCATCTGATGCTCAAGCCACACAGCTTATAAGCCACGCGTTCAGTGACGCTTCTAGCACTCGTGGTGGTGCCAACTTcc                                                                                                                                            >  1:289196/1‑301 (MQ=255)`  
                                                                                                                                                                     `gATTATCAGCGTGGTTTCAACGGGGACATTACCGAGCGTAACATCTCGCTGTATGGTGCGCATGATAACTTCTTTAGCCAGCAAGCTCAGAAGGGCGCTATCATGAACAGCCGAGTGGAACTCAACGGTGTCCTTCAAGACCCTGATATGCTGCGTCGTCTAGACTCTGCTGGCTTCTTTGAGAAGTATATCGACAACTGTCTGGTTACTGGCGCAATCCCATCTGATGCTCAAGCCACACAGCTTATAAGCCAAGCGTTCAGTGACGCTTCTAGCCGTGCTGGTGGTGCTGACTTCCtta                                                                                                                                         >  1:76788/1‑299 (MQ=255)`  
                                                                                                                                                                       `ttATCAGCGTGGTGTAAACGGGGAAATTACCGAGCGTAACATCTCGCTGTATGGTGCGCATGATAACTTCTTGAGCCAGCAAGCTCAGAAGGGCGCTATCATGAACAGCCGAGTGGAACTCAACGGTGTCCTTCAAGACCCTGATATGCTGCGTCGTCCAGACTCTGCTGACTTCTTTGAGAAGTATATCGACAACGGTCTGGTTACTGGCGCAATCCCATCTGATGCTCAAGCCACACAGCTTATAAGCCAAGCGTTCAGTGACGCTTCTAGCCGTGCTGGTGGTGCTGACTTCCTGATg                                                                                                                                       <  1:382458/301‑1 (MQ=255)`  
                                                                                                                                                                                          `acagACATTACCGAGCGTAACATCTCGCTGTATGGTGCGCATGATAACTTCTTGAGCCAGCAAGCTCAGAAGGGCGCTATCATGAACAGCCGAGTGGAACTCAACGGTGTCCTTCAAGACCCTGATATGCTGCGTCGTCCAGACTCTGCTGACTTCTTTGAGAAGTATATCGACAACGGTCTGGTTACTGGCGCAATCCCATCTGATGCTCAAGCCACACAGCTTATAAGCCAAGCGTTCAGTGACGCTTCTAACCGTGCTGGTGGTGCTGACTTCCTGATGCGAGTCGGTGACAAGAAgg                                                                                                                    <  1:375370/298‑1 (MQ=255)`  
                                                                                                                                                                                           `gggagatgtgtataagagacacCTCGCTGTATGGTGCGCATGATACCTTCTTGAGCCAGCAAGCTCAGAAGGGCGCTATCATGAACAGCCGAGTGGAACTCAACGGTGTCCTTCAAGACCCTGATATGCTGCGTCGTCCAGACTCTGCTGACTTCTTTGAGAAGTATATCGACAACGGTCTGGTTACTGGCGCAATCCCATCTGATGCTCAAGCCACACAGCTTATAAGCAAAGCGTTCAGTGACGCTTCTAGCCGTGCTGGTGGTGCTGACTTCCTGATGCGAGTCGGTGACAAGAAGGt                                                                                                                   <  1:391501/283‑1 (MQ=255)`  
                                                                                                                                                                                           `gggACATTACCGAGCGTAACATCTCGCTGTATGGTGCGCATGATAACTTCTTGAGCCAGCAAGCTCAGAAGGGCGCTATCATGAACAGCCGCGTGGAACTCAACGGTGTACTTAAAGACCCTGATATGCTGCGTCGTCCAGACTCTGCTGACTTCTTTGAGAAGTCTATCGCCAACGTTCTGGTTACTGGCGCAATCCCATCTGATGCTCAAGCCACACAGCTTATAAGCCAAGCGTTCATTGACGCTTCTAGCCGTGCTGGTTGTGCTGACTTCCTGATGCGCGTCGTTGACAAGAATGt                                                                                                                   >  1:43548/1‑301 (MQ=255)`  
                                                                                                                                                                                            `ggACATTACCGAGCGTAACATCTCGCTGTATGGTGCGCATGATAACTTCTTGAGCCAGCAAGCTCAGAAGGTCGCTATCATGAACAGCCGAGTGGAACTCAACGGTGTCCTTCAAGACCCTGATATGCTGCGTCGTCCAGACTCTGCTGACTTCTTTGAGCAGTATATCGCTAACGGTCTGGTTACTGGCTCCATCCCATCTGATGCTCAAGCCACACAGCTTATTAGCCAAGCGTTCAGTGCCGCTTCTAGCCGTGCTGGTGATTCTGACTTGCTGATCCGAGTAGGTGACAAGTAGGTa                                                                                                                  >  1:263757/1‑301 (MQ=255)`  
                                                                                                                                                                                              `gcATTACCGAGCGTAACATCTCGCTGTATGGTGCGCATGATAACTTCTTGAGCCAGCAAGCTCAGAAGGGCGCTATCATGAACATCCGAGTGGAACTCAACGGTGTCCTTCAAGACCCTGATATGCTGCGTCGTCCAGACTCTGCTGACTTCTTTGAGAAGTATATCGACAACGGTCTGGTTACTGGCGCACTCCCATCTGATGCTCAAGCCACACAGCTTATAAGCCAAGCGTTCAGTGACGCTGCTAGCCGTGCTGGTGGTGCTGACTTCCTGATGCGAGTCGGTGACAAGAAGGTaaa                                                                                                                >  1:86358/2‑300 (MQ=255)`  
                                                                                                                                                                                                `aTTACCGAGCGTAACATCTCGCTGTATGGTGCGCATGATAACTTCTTGAGCCAGCAAGCTCAGAAGGGCGCTATCATTAATAGCCGAGTGGAACTCAACGGTGTACTTCAAGACCCTGATATGCTGCGTCCTCCAGACTCTGCTGACTTCTTTGAGAAGTATATCGACAACGGTCTGGTTACTGGCGCAATCCCATCTGATGCTCAAGCCACACAGCTTATAAGCCAAGCGTTCAGTGACGCTTCTAGCCGTGCTGGTGGTGCTGACTTCCTGATGCGAGTCGGTGACAAGAAGGTAacac                                                                                                              <  1:13316/301‑1 (MQ=255)`  
                                                                                                                                                                                                `aTTACCGAGCGTAACATCGCGCTGTATGGTGCGCATGATAACTTATTGAGCCAGCAAGCTCAGAATGGCGCTATCATGAACAGCCGAGTGGAACTAAACGGTGTCCTTCAAGACCCTGATATGCTGCGTCGTCCAGACTCTGCTGACTTCTTTGAGAAGTATATCGACAACGGTCTGGTTACGGGCGAAATCCCATATGATGCTCAAGCCACACAGCTTATAAGCCAAGCGTTCAGTGACGCGTCTAGCCGTGCTGGTGGTGCTGACTTCCTGATGCGAGTCGGTGACAAGAAGGTAacac                                                                                                              <  1:2369/301‑1 (MQ=255)`  
                                                                                                                                                                                                       `aGCGTAACATCTCGCTGTATGGTGCGCAGGATAACTTCTTGAGCCAGCAAGCTAAGAAGGGCGCTATCATGAACAGCCGAGTGGAACTCAACGGTGTCCTTCAAGACCCTGATATGCTGCGTCGTCCAGACTCTGCTGACTTCTTTGAGAAGTATATCGACAACGGTCTGGTTACTGGCGCAATCCCATCTGATGCTCAAGCCACACAGCTTATAAGCCAAGCGTTCAGTGACGCTTCTAGCCGTGCTGGTGGTGCTGACTTCCTGATGCGAGTCGGTGACAAGAAGGTAACACTTAACgg                                                                                                       <  1:464478/301‑1 (MQ=255)`  
                                                                                                                                                                                                             `aCATCTCGCTGTATGGTGCGCATGATAACTTCTTGAGCCAGCAAGCTCAGAAGGGCGCTATCATGAACAGCCGAGTGGAACTCAACGGTGTCCTTCAAGACCCTGATATGCTGCGTCGTCCAGACTCTGCTGACTTCTTTGAGAAGTATATCGACAACGGTCTGGTTACTGGCGCAATCCCATCTGATGCTCAAGCCACACAGCTTATAAGCCAAGCGTTCAGTGACGCTTCTAGCCGTGCTGGTGGTGCTGACTTCCTGATGCGAGTCGGTGACAAGAAGGTAACACTTAACggctgtct                                                                                                 >  1:315031/1‑295 (MQ=255)`  
                                                                                                                                                                                                               `ctctcGCTGTATGGTGCGCATGATAACTTCTTGAGCCAGCAAGCTCAGAAGGGCGCTATCATGAACAGCCGAGTGGAACTCAACGGTGTCCTTCAAGACCCTGATATGCTGCGTCGTCCAGACTCTGCTGACGTCTTTGAGAAGTATATCGACAACGGTCTGGTTACTGGCGCAATCCCATCTGATGCTCAAGCCACACAGCTTATAAGCCAAGCGTTCAGTGACGCTTCTAGCCGTGCTGGTGGTGCTGACTTCCTGATGCGAGTCGGTGACAAGAAGGTAACACTTAACGGAGCCACTa                                                                                               <  1:143404/300‑1 (MQ=255)`  
                                                                                                                                                                                                                   `cGCTGTATGGTGCGCATGATAACTTCTTGAGCCAGCAAGCTCAGAAGGGCGCTATCATGAACAGCCGAGTGGAACTCAACGGTGTCCTTCAAGACCCTGATATGCTGCGTCGTCCAGACTCTGCTGACTTCTTTGAGAAGTATATCGACAACGGTCTGGTTACTGGCGCAATCCCATCTGATGCTCAAGCCACACAGCTTATAAGCCAAGCGTTCAGTGACGCTTCTAGCCGTGCTGGTGGTGCTGACTTCCTGATGCGAGTCGGTGACAAGAAGGTAACACTTAACGGAGCCACTACgct                                                                                           >  1:170547/1‑299 (MQ=255)`  
                                                                                                                                                                                                                   `cGCTGTATGGTGCGCATGATAACTTCTTGAGCCAGCAAGCTCAGAAGGGCGCGATCATGAACAGCCGAGTGGAACTCAACGGTGTCCTTCAAGACCCTGATATGCTGCGTCGTCCAGACTCTGCTGACTTCTTTGAGAAGTATATCGACAACGGTCTGGTTACTGGCGCAATCCCATCTGATGCTCAAGCCACACAGCTTATAAGCCAAGCGTTCAGTGACGCTTCTAGCCGTGCTGGTGGTGCTGACTTCCTGATGCGAGTCGGTGACAAGAAGGTAACACTTAACGGAGCCACTACGAc                                                                                           <  1:403787/301‑1 (MQ=255)`  
                                                                                                                                                                                                                    `gCTGTATGGTGCGCATGATAACTTCTTGAGCCAGCAAGCTCAGAAGGGCGCTATCATGAACAGCCGAGTGGAACTCAACGGTGTCCTTCAAGACCCTGATATGCTGCGTCGTCCAGACTCTGCTGACTTCTTTGAGAAGTATATCGACAACGGTCTGGTTACTGGCGCAATCCCATCTGATGCTCAAGCCACACAGCTTATAAGCCAAGCGTTCAGTGACGCTTCTAGCCGTGCTGGTGGTGCTGACTTCCTGATGCGAGTCGGTGACACGAAGGTAACACTTCACggctgtccatcatac                                                                                          >  1:499862/1‑288 (MQ=255)`  
                                                                                                                                                                                                                         `attgcgcacATGATAAATGCTTGAGCCAGCAAGCTCAGAAGGGCGCTATCATGAACAGCCGAGTGGATCTCAACGGTGTCCTTCAAGACCCTGATATGCTGCGTCGTCCAGACTCTGCTGACTTCTTTGAGAAGTATATCGACAACGGTCTGGTTACTGGCGCAATCCCATCTGATGCTCAAGCCACACAGCTTATAAGCCAAGCGTTCAGTGACGCTTCTAGCCGTGCTGGTGGTGCTGACTTCCTGATGCGAGTCGGTGACAAGAAGGTAACACTTAACGGAGCCACTACGACTTACCg                                                                                     <  1:309620/293‑1 (MQ=255)`  
                                                                                                                                                                                                                         `aTGGTGCGCATGATAACTTCTTGAGCAAGCAAGCTCAGAAGGGCGCTATAATGAACAGCCGAGTGGAACTCAACGGTGTCCTTAAAGACCCTGATATGCTGCGTCGTCCAGACTCTGCTGACTTCTTTGAGAAGTATATCGACAACGGTCTGGTTACTGGCGCAATCCCATCTGATGCTCAAGCCACACAGCTTATAAGCCAAGCGTTCAGTGACGCTTCTAGCCGTGCTGGTGGTGCTGACTTCCTGATGCGAGTCGGTGACAAGAAGGTAACACTTAACGGAGCCACTACGACTTACCg                                                                                     <  1:149972/301‑1 (MQ=255)`  
                                                                                                                                                                                                                             `tGCGCATGATAACTTCTTGGGCCAGCAAGCTCAGAAGGGCGCTATCATGAACAGCCGAGTGGAACTCAACGGTGTCCTTCAAGACCCTGATATGCTGCGTCGTCCAGACTCTGCTGACTTCTTTGAGAAGTATATCGACAACGGTCTGGTTACTGGCGCAATCCCATCTGATGCTCAAGCCACACAGCTTATAAGCCAAGCGTTCAGTGACGCTTCTAGCCGTGCTGGTGGTGCTGACTTCCTGATGCGAGTCGGTGACAAGAAGGTAACACTTAACGGAGCCACTACGACTTACCGagag                                                                                 <  1:415575/301‑1 (MQ=255)`  
                                                                                                                                                                                                                                 `cATGATAACTTCTTGAGCCAGCAAGCTCAGAAGGGCGCTATCATGAACAGCCGAGTGGAACTCAACGGTGTCCTTCAAGACCCTGATATGCTGCGTCGTCCAGACTCTGCTGACTTCTTTGAGAAGTATATCGACAACGGTCTGGTTACTGGCGCAATCCCATCTGATGCTCAAGCCACACAGCTTATAAGCCAAGCGTTCAGTGACGCTTCTAGCCGTGCTGGTGGTGCTGACTTCCTGATGCGAGTCGGTGACAAGAAGGTAACACTTAACGGAGCCACTACGACTTACCGAGAGTTGa                                                                             >  1:460367/1‑301 (MQ=255)`  
                                                                                                                                                                                                                                   `tGATAACTTCTTGAGCCAGCAAGCTCAGAATGGCGCTATCATTAACAGCCGAGTGGAACTCCACGGTGTCCTTCAAGACCCTGATATTCTGCGTCGTCCAGAATCTGGTGACTTCTTTGAGAAGTATATCCACAACGGTCTGGTTACTGACGCAATCCCATCTGATGCTCAAGCCACACAGCTTATAAGCCAAGCGTTCAGTGACGATTCTAGTCCTGCTGGTGGAGCAGACTTCATGAATCGAGTCGGTGTCAAGAAGCAAACCATTAACGCACCCACttcccatcacagacaagcgtat                                                                           >  1:495612/1‑280 (MQ=255)`  
                                                                                                                                                                                                                                   `tGATAACTTCTTGAGCCAGCAAGCTCAGAAGGGCGCTATCATGAACAGCCGAGTGGAACTCAACGGTGTCCTTCAAGACCCTGATATGCTGCGTCGTCCAGACTCTGCTGACTTCTTTGAGAAGTATATCGACAACGGTCTGGTTACTGGCGCAATCCCATCTGATGCTCAAGCCACACAGCTTATAAGCCAAGCGTTCAGTGACGCTTCTAGCCGTGCTGGTGGTGCTGACTTCCTGATGCGAGTCGGTGACAAGAAGGTAACACTTAACGGGGCCACTACGACTTACCGGGAGTTGAtt                                                                           >  1:15442/1‑301 (MQ=255)`  
                                                                                                                                                                                                                                   `tGATAACTTCTTGAGCCAGCAAGCTCAGAAGGGCGCTATCATGAACAGCCGAGTGGAACTCAACGGTGTCCTTCAAGACCCTGATATGCTGCGTCGTCCAGACTCTGCTGACTTCTTTGAGAAGTATATCGACAACGGTCTGGTTACTGGCGCAATCCCATCTGATGCTCAAGCCACACAGCTTATAAGCCAAGCGTTCAGTGACGCTTCTAGCCGTGCTGGTGGTGCTGACTTCCTGATGCGAGTCGGTGACAAGAAGGTAACACTTAACGGAGCCACTACGACTTACCGAGAGTTGAtt                                                                           <  1:430861/301‑1 (MQ=255)`  
                                                                                                                                                                                                                                     `aTAACTTCTTGAGCCAGCAAGCTCAGAAGGGCGCGATCATGAACAGCCGAGTGGAACTCAACGGTGTCCTTAAAGACCCTGATATGCTGCGTCGTCCAGACTCTGCTGACTTCTTTGAGAAGTATATCGACAACGGTCTGGTTACTGGCGCAATCCCATCTGATGCTCAAGCCACACAGCTTATAAGCCAAGCGTTCAGTGACGCTTCTAGCCGTGCTGGTGGTGCTGACTTCCTGATGCGAGTCGGTGACAAGAAGGTAACACTTAACGGAGCCACTACGACTTACCGAGAGTTGATTgg                                                                         <  1:206178/301‑1 (MQ=255)`  
                                                                                                                                                                                                                                       `aaCTTCTTGAGCCAGCAAGCTCAGAAGGGCGCTATCATGAACAGCCGAGTGGAACTCAACGGTGTCCTTCAATACCCTGATATGCTGCGTCGTCCAGACTCTGCTGACTTCTTTGAGAAGTATATCGACAACGGTCTGGTTACTGGCGCAATCCCATCTGATGCTCAAGCCACACAGCTTATAAGCCAAGCGTTCAGTGACGCTTCTAGCCGTGCTGGTGGTGCTGACTTCCTGAGACGAGTCGGTGACAAGAAGGTAACACTTAACGGAGCCACTACGACTTACCGAGAGtttactctgt                                                                       >  1:482582/1‑293 (MQ=255)`  
                                                                                                                                                                                                                                             `ttGGGCCAGCAAGCTCAGAATGGCGCTATCATTAACAGCCGAGTGGAACTCAACGGTGTCCTTCAAGACCCTGATATGCTGCGTCGTCCAGACTCTGCTGACTTCTTTGAGAAGTATATCGACAACGGGCTGGTTACTGGCGCAATCCCATCTGATGCTCAAGCCACACAGCTTATAAGCCAAGCGTTCAGTGACGCTTCTAGCCGTGCTGGTGGTGCTGACTTCCTGATGCGAGTCGATGACAAGAAGGTAACACTTAACGGAGCCACTACGACTTACCGAGAGTTGATTGGTGAGGAAc                                                                 <  1:58213/301‑1 (MQ=255)`  
                                                                                                                                                                                                                                                  `ccAGCAAGCTCAGAAGGGCGCTATCATGAACAGCCGAGTGGAACTCAACGGTGTCCTTAAAGACCCTGATATGCTGCGTCGTCCAGACTCTGCTGACTTCTTTGAGAAGTATATCGACAACGGTCTGGTTACTGGCGCAATCCCATCTGATGCTCAAGCCACACAGCTTATAAGCCAAGCGTTCAGTGACGCTTCTAGCCGTGCTGGTGGTGCTTACTTCCTGATGCGAGTCGGTGACAAGAAGGTAACACTTAACGGAGCCACTACGACTTACCGAGAGTTGATTGGTGAGGAACAGTgg                                                            <  1:474253/301‑1 (MQ=255)`  
                                                                                                                                                                                                                                                         `gCTCAGAAGGGCGCTATCATGAACAGCCGAGTGGAACTCAACTGTGTCCTTAAAGACCCTGATATGCTGCTTCGTCCAGACTCTGCTGACTTCTTTGAGAAGTATATCGACAACGGTCTGGTTACTGGCGCAATCCCATCTGATGCTCAAGCCACACAGCTTATAAGCCAAGCGTTCAGTGACGCTTCTAGCCGTGCTGGTGGTGCTGACTTCCTGATGCGAGTCGGTGACAAGTAGGTAACACTTAACGGCGCCACTACGACTTACCGAGAGTTGATTGGTGAGGAACAGTGGAACGctt                                                     >  1:445972/1‑300 (MQ=255)`  
                                                                                                                                                                                                                                                         `gCTCAGAAGGGCGCTATCATGAACAGCCGAGTGGAACTCAACGGTGTCCTTAAAGACCCTGATATGCTGAGTCGTCCAGACTCTGCTGACTTCTTTGAGAAGTATATCGACAACGGTCTGGTTACTGGCGCAATCCCATCTGATGCTCAAGCCACACAGCTTATAAGCCAAGCGTTCAGTGACGCTTCTAGCCGTGCTGGTGGTGCTGACTTCCTGATGCGAGTCGGTGACAAGAAGGTAACACTTAACGGAGCCACTACGACTTACCGAGAGTTGATTGGTGAGGAACCGTGGAACgtcc                                                     >  1:426917/1‑298 (MQ=255)`  
                                                                                                                                                                                                                                                            `tataagagacagaTCATGAACAGCCGAGTGGAACTCAACGGTGTCCTTCAAGACCCTGATATGCTGCGTCGTCCAGACTCTGCTGACTTCTTTGAGAAGTATATCGACCACGGTCTGGTTACTGGAGCAATCCCATCTGATGCTCAAGCCACACAGCTTATAAGCCAAGCGTTCAGTGACGCTTCTAGCCGTGCTGGTGGTGCTGACTTCCTGATGCGAGTCGGTGACAAGAAGGTAACACTTAACGGAGCCACTACGACTTACCGAGAGTTGATTGGTGAGGAACAGTGGAACGCTCTCa                                                  <  1:387486/289‑1 (MQ=255)`  
                                                                                                                                                                                                                                                            `cAGAAGGGCGCTATCATGAACAGCCGAGTGGAACTCAACGGTGTCCTTCAAGACCCTGATATGCTGCGTCGTCCAGACTCTGCTGACTTCTTTGAGAAGTATATCGACAACGGTCTGGTTACTGGCGCAATCCCATCTGATGCTCAAGCCACACAGCTTATAAGCCAAGCGTTCAGTGACGCTTCTAGCCGTGCTGGTGGTGCTGACTTCCTGATGCGAGTCGGTGACAAGAAGGTAACACTTAACGGAGCCACTACGACTTACCGAGAGTGGATTGGTGAGTAACCGGGGCACGCTCTCa                                                  >  1:72463/1‑301 (MQ=255)`  
                                                                                                                                                                                                                                                                 `gggCGCTATCATGTACAGCCGTGTGGAACTCAACGTTTTCCTTCAAGACCCTGAGATTCTGCGTCTGCCAGTCTCTGCTTACTTCTTTGAAAATTATATCGACACCGGTCTGGTTACTGGCGCAATCCCATCTGATGCTCAAGCCACTCAGCTTATAAGCCAAGCGTTCAGTGACGCTTCTAGCCGTGCTGGTGGTGCTGACTTCCTTATGCGAGTCGGTGACAAGAAGGTAACACTTAACGGAGCCACTACGACTTACCGAGAGTTGATTGGTGAGGAACAGTGGAACGCTCTCATGGTc                                             <  1:455666/301‑1 (MQ=255)`  
                                                                                                                                                                                                                                                                        `aTCATTAACATCCGAGTGGAACTCAACGGTGTCCTTCAAGACCCTGATATGCTGCGTCGTCCAGACTCTGCTGACTTCTTTGAGAAGTATATCGACAACGGTCTGGTTACTGGCGCAATCCCATCTTATGCTCAAGCCACACACCTTATAAGCCACGCGTTCAGTGACGCTTCTAGCCGTTCTGGTGGTGCTGACTTCCTGATGCGAGTCGGCGTCCAGAAGGTAATACATAACTGAGCCATTACGACTTACCGAGCGTCGTTTGGAGAGCAACAGTGGAATGCTCCCAAGGTCACAGcac                                      >  1:371086/1‑301 (MQ=255)`  
                                                                                                                                                                                                                                                                        `aTCATGAACAGCCGAGTGGAACTCAACGGTGTCCTTCAAGACCCTGATATGCTGCGTCGTCCAGACTCTGCTGACTTCTTTGAGAAGTATATCGACAACGGTCTGGTTACTGGCGCAATCCCATCTGATGCTCAAGCCACACAGCTTATAAGCCAAGCGTTCAGTGACGCTTCTAGCCGTGCTGGTGGTGCTGACTTCCTGATGCGAGTCGGTGACAAGAAGGTAACACTTAACGGAGCCACTACGACTTACCGAGAGTTGATTGGTGAGGAACAGTGGAACGCTCTCATGGTCACAGcac                                      >  1:210390/1‑301 (MQ=255)`  
                                                                                                                                                                                                                                                                          `cATGAACAGCCGAGTGGAACTCAACGGTGTCCTTAAAGACCCTGATATGCTGCGTCGTCCAGACTCTGCTGACTTCTTTGAGAAGTATATCGACAACGGTCTGGTTACTGGCGAAATCCCATCTGATGCTCAAGCCACACAGCTTATAAGCCAAGCGTTCAGTGACGCTTCTAGCCGTGCTGGTGGTGCTGACTTCCTGATGCGAGTCGGTGACAAGAAGGTAACACTTAACGGAGCCACTACGACTTACCGAGAGTTGATTGGTGAGGAACAGTGGAACGCTCTCATGGTCACAGCACaa                                    >  1:283133/1‑301 (MQ=255)`  
                                                                                                                                                                                                                                                                           `aggAACAGCCGAGTGGAACTCAACGGTGTCCTTCAATACCCTGATATGCTGCGTCGTCCAGACTCTTCTGACTTCTTTGAGAAGTATATCGACAACGGTCTGGTTACTGGCGCAATCCCATCTGATGCTCAAGCCACACAGCTTATAAGCCAAGCGTTCAGTGACGCTTCTAGCCGTGCTGGTGGTGCTGACTTCCTGATGCGAGTCGGTGACAAGAAGGTAACACTTAACGGAGCCACTACGACTTACCGAGAGTTGATTGGTGAGGAACAGTGGAACGCTCTCATGGTCACAGCACAAc                                   <  1:468209/299‑1 (MQ=255)`  
                                                                                                                                                                                                                                                                                 `cgcggagaagcatagaaaggacagcTTCAAGCCCCTGATATACTGCGTCGTCCAGACTCTGCTGACTTCTTTGAGAAATATATCGACAACGGTCTGGTTACTGGCGCAATCCCATCTGATGCTCAAGCCACACAGCTTATAAGCCAAGCGTTCGGTGACGCTTCTAGCCGTGCTGGTGGTGCTGACTTCCTAATGCGAGTCGGTGACAAAAAGGTAACACTTAACGAAGCCACTACGACTTACCGAGAATTGGTTGGTGAGGAACAGTGGAACGCTCTCATGGTCACAGCACAACGTtctc                             <  1:166218/277‑1 (MQ=255)`  
                                                                                                                                                                                                                                                                                 `aagagacaggaactaaaggggacacTTCAAGACCCTGATATGCTGCGTCGTCCAGACTCTGCTGACTTCTTTGCGAAGTATATCGATAACGGGTTGGTTACTGGCGCAATCCCATCTGATGGTAAAGCCACACAGCTTAGAAGCCAAGCGTGCAGCGACGCTTCTAGCCGTGCTGGTTGTGCTGACTTCCGGATGCGAGTCGGTGACAAGAAGGTAACACTTAACGGAGCCACTACGACTTACCGAGAGTTGATTGGTGAGGAACAGTGGAACGCTCTCATGGTCACAGCACAACGTtctc                             <  1:366415/277‑1 (MQ=255)`  
                                                                                                                                                                                                                                                                                                   `ggTGTCCTTCAAGACCCTGATATGCTGCGTCGTCCTGACTCTGCTGACTTCTTTGAGAAGTATATCGACAACGGTCTGGTTACTGGCGAAATCCCATCTGATGCTCAAGCCACACAGCTTATAAGCCAAGCGTTCAGTGACGCTTCTAGCCGTGCTGGTGGTGCTGACTTCCTGATGCGAGTCGGTGACAAGAAGGTAACACTTAACGGAGCCACTACGACTTACCGAGAGTTGATTGGTGAGGAACAGTGGAACGCTCTCATGGTCACAGCACAACGTTCTCAGTTTGAGACTGACGCGa           <  1:214096/301‑1 (MQ=255)`  
                                                                                                                                                                                                                                                                                                      `gtCCTTCAAGACCCTGATATGCTGCGTCGTCCAGACTCTGCTGACTTCTTTGAGAAGTATATCGACAACGGTCTGGTTACTGGCGCAATCCCATCTGATGCTCAAGCCACACAGCTTATAAGCCAAGCGTTCAGTGACGCTTCTAGCCGTGCTGGTGGTGCTGACTTACTGATGCGAGTCGGTGACAAGAAGGTAACACTTAACGGAGCCACTACGACTTACAGAGAGTTGATTGGTGAGGAACAGTGGAACGCTCTCATGGTCACAGCACAACGTTCTCAGTTTGAGACTGACGCGAAGc        >  1:41168/1‑301 (MQ=255)`  
                                                                                                                                                                                                                                                                                                         `cTTCAAGACCCTGATATGCTGCGTCGTCCAGACTCTGCTGACTTCTTTGAGAAGTATATCGACAACGGTCTGGTTACTGGCGCAATCCCATCTGATGCTCAAGCAACACAGCTTATAAGCCAAGCGTTCAGTGACGCTTCTAGCCGTGCTGGTGGTGCTAACTTCCTGATGCCAGTCGGGGACAAGAAGGTAACACTTAACGGAGCCACTACGACTTGCCGAGAGTTGATTTGTGAGGAACAGTGGAACGCTCTCATGGTCCCAGAACAACGTTCTCAGTTTGAGACTGACGCGACGCTGa     >  1:103418/1‑301 (MQ=255)`  
                                                                                                                                                                                                                                                                                                           `tCAAGACCCTGATATGCTGCGTCGTCCATACTCTGCTTACTTCTTTGAGAAGTATATCGACAACGGTCTGGTTACTGGCGCAATCCCATCTGATGCTCAAGCCACACAGCTTATAAGCCAAGCGTTCAGTGACGCTTCTAGCCGTGCTGGTGGTGCTGACTTCCTGATGCGAGTCGGTGACAAGAAGGTAACACTTAACGGAGCCACTACGACTTACCGAGAGTTGATTGGTGAGGAACAGTGGAACGCTCTCATGGTCACAGCACAACGTTCTCAGTTTGAGACTGACGCGAAGCTGAAc   <  1:268218/301‑1 (MQ=255)`  

                                                                                                                                                                                                                                                                                                            `|`                                                                                                                                                                                                                                                                                                              
`AAGACTGGTCGTAACGCTGCGTATCTTGTGGACGATGACGTTATGCAGAAGATAAAAGAGGGTGTCTTCCGTACTCGCGAAGAGATGGAAGAGTATCGCCATAGTCGCCTTCAAGAGGGCGCTAAGGTATACGCTGAGCAGTTCGGCATCGACCCTGAGGACGTTGATTATCAGCGTGGTTTCAACGGGGACATTACCGAGCGTAACATCTCGCTGTATGGTGCGCATGATAACTTCTTGAGCCAGCAAGCTCAGAAGGGCGCTATCATGAACAGCCGAGTGGAACTCAACGGTGTCCTTCAAGACCCTGATATGCTGCGTCGTCCAGACTCTGCTGACTTCTTTGAGAAGTATATCGACAACGGTCTGGTTACTGGCGCAATCCCATCTGATGCTCAAGCCACACAGCTTATAAGCCAAGCGTTCAGTGACGCTTCTAGCCGTGCTGGTGGTGCTGACTTCCTGATGCGAGTCGGTGACAAGAAGGTAACACTTAACGGAGCCACTACGACTTACCGAGAGTTGATTGGTGAGGAACAGTGGAACGCTCTCATGGTCACAGCACAACGTTCTCAGTTTGAGACTGACGCGAAGCTGAACG  >  NC_001604.1/28643‑29243`

  

Alignment Legend

Aligned base mismatch/match (shaded by quality score): `ATCG/ATCG` `< 3 ≤` `ATCG/ATCG` `< 9 ≤` `ATCG/ATCG` `< 15 ≤` `ATCG/ATCG` `< 31 ≤` `ATCG/ATCG` `< 38 ≤` `ATCG/ATCG`

Unaligned base: `atcg`    Masked matching base: `atcg`    Alignment gap: `‑`    Deleted base: `‑`
